# Management of Acute Kidney Injury in Extremely Low Birth Weight Infants

**DOI:** 10.3389/fped.2022.867715

**Published:** 2022-03-30

**Authors:** Aoife Branagan, Caoimhe S. Costigan, Maria Stack, Cara Slagle, Eleanor J. Molloy

**Affiliations:** ^1^Paediatrics, Trinity Research in Childhood Centre (TRICC), Trinity College Dublin, Dublin, Ireland; ^2^Neonatology, Coombe Women's and Infants University Hospital, Dublin, Ireland; ^3^Nephrology, Children's Health Ireland (CHI) at Crumlin & Temple Street, Dublin, Ireland; ^4^Division of Neonatology & Pulmonary Biology and the Center for Acute Care Nephrology, Cincinnati Children's Hospital Medical Center, Cincinnati, OH, United States; ^5^The University of Cincinnati College of Medicine, Cincinnati, OH, United States; ^6^Children's Hospital Ireland (CHI) at Tallaght, Dublin, Ireland; ^7^Neonatology, Children's Health Ireland (CHI) at Crumlin, Dublin, Ireland

**Keywords:** preterm infant, extremely low birth weight, renal biomarkers, hypertension, acute kidney injury

## Abstract

Acute kidney injury (AKI) is a common problem in the neonatal intensive care unit (NICU). Neonates born at <1,000 g (extremely low birth weight, ELBW) are at an increased risk of secondary associated comorbidities such as intrauterine growth restriction, prematurity, volume restriction, ischaemic injury, among others. Studies estimate up to 50% ELBW infants experience at least one episode of AKI during their NICU stay. Although no curative treatment for AKI currently exists, recognition is vital to reduce potential ongoing injury and mitigate long-term consequences of AKI. However, the definition of AKI is imperfect in this population and presents clinical challenges to correct identification, thus contributing to under recognition and reporting. Additionally, the absence of guidelines for the management of AKI in ELBW infants has led to variations in practice. This review summarizes AKI in the ELBW infant and includes suggestions such as close observation of daily fluid balance, review of medications to reduce nephrotoxic exposure, management of electrolytes, maximizing nutrition, and the use of diuretics and/or dialysis when appropriate.

## Introduction

Acute kidney injury (AKI) is a common complication in infants requiring intensive care. AKI in the newborn period has been associated with an increase in morbidity and mortality (1). Those born preterm (<37 weeks gestation), especially those born at extremely premature gestation (<28 weeks gestation) are at additional risk for AKI compared to term infants and older populations, with studies reporting up to 48% incidence in AKI in those under 28 weeks gestation (1). Nephrogenesis is not complete until about 36 weeks gestation and may contribute to this increased risk.

Preterm infants, especially extremely low gestational age neonates (ELGAN) (<28 weeks gestation) and/or extremely low birth weight (ELBW) neonates (<1,000 g) offer unique challenges in the diagnosis and management of AKI. AKI occurs at comparable levels in this population to critically ill adults and children (1). In addition to incomplete structural maturity these neonates demonstrate impaired functional renal maturity. Serum creatinine (sCr) is suboptimal biomarker for AKI in neonates secondary to the influences of maternal sCr in the first days of life, muscle mass, nutritional status, and bilirubin levels (associated with type of assay used). Compounding this, AKI was long thought to be reversible, thus delaying progression of research, clinical recognition, and ultimately expansion of renal replacement therapy (RRT) options. Exploration and research into treatment options is limited and RRT options in this population are largely restricted (2–4).

## Renal Development

Glomeruli formation begins in the 5th week of gestation. The production of urine then follows, commencing during the 9th week of gestation (5). The passage of urine aids in accumulation of amniotic fluid and is critical for pulmonary development (5). Nephrogenesis is complete by ~34 weeks gestation (6). Premature birth disrupts this developmental process; however evidence supports post-natal nephrogenesis to 36 weeks corrected gestational age (GA). However, post-natal development of nephrons exhibit abnormal nephron morphology and function (3, 4). Any episodes of AKI may further impact glomerulogenesis (3). Incomplete and abnormal nephrogenesis contributes to a nephron deficit in preterm infants and are thought to increase vulnerability of ongoing impaired renal function (4, 7).

Functional maturity of the kidney does not fully develop until 2 years of age but increases most profoundly in the first week of life and is irrespective of birth GA (8). Glomerular filtration rate (GFR) will rise from 5 to 40 mL/min/1.73 m^2^ during the first week as a result of a substantial increase in cardiac output received by the fetus. During pregnancy, the fetal kidney receives ~3% of cardiac output which rises to 10% by the end of the first week of life, and then more slowly. GFR reaches 65 ml/min by 2 months and ultimately adult levels of 120 ml/min by 2 years of age when the kidney receives ~25% of cardiac output (2). The increase in GFR and renal blood flow overtime is also assisted by a neuro-endocrine mediated decrease in renal vascular resistance (2, 9).

Nephron number *in vivo* is difficult to measure and intrauterine growth restriction (IUGR), preterm birth (PTB) and low birth weight (LBW) remain the best clinical surrogates as these patients are more likely to have a lower nephron number (10). PTB and infants of LBW are increasing with the global risks of 10 and 15%, respectively, therefore millions of children are born at risk of chronic kidney disease (CKD) (10, 11). In the United States in adolescents born at LBW, there was a 1 in 13 incidence of reduced GFR and similar risk of increased blood pressure progressing with age (7, 12). Chronic kidney disease (CKD) may not occur just due to a low nephron number alone but may be more vulnerable to additional renal injury (10).”

The renal tubular system, responsible for the regulation of water and electrolyte excretion and reabsorbtion, is also dependant on gestational age at birth with an ability to increase functional ability in the postnatal period. A transient “tubulapathy of prematurity” is recognized, describing renal tubular immaturity along with a decreased response to aldosterone, inability to adequately handle free water, electrolytes, small proteins and bicarbonate resulting in an inability to appropriately concentrate urine, metabolic acidosis, electrolyte imbalance, and poor growth (13).

## Definitions of AKI

AKI is characterized by increased sCr and nitrogenous waste products, a fall in GFR and the inability of the kidney to accurately regulate the fluid and electrolyte homeostasis of the infant and older cohorts (9). Multiple definitions have been used to define neonatal AKI until recently when consensus groups prioritized standardizing the definition to progress the field.

The neonatal modified KDIGO (Kidney Disease—Improving Global Outcome) criteria (Table 1) were suggested to define AKI and is supported by the National Institute for Health (NIH) (14). The neonatal modified KDIGO (nKDIGO) definition relies on an increase in sCr from a previously measured trough and divides neonatal AKI into 3 stages of increasing severity (Table 1). The nKDIGO criteria independently predict clinical outcomes (15). A NIH National Institute of Diabetes, Digestive, and Kidney Diseases (NIDDK) neonatal AKI definition working group found it to be the best approach currently available (5).

**Table 1:** Modified KDIGO (Kidney disease—improving global outcomes) criteria and Modified Neonatal KDIGO criteria.

**Stage**	**Change in serum creatinine**	**Other accepted criteria**	**Urine output**
No AKI	No change or Rise of <0.3 mg/dl	No	>1 ml/kg/h
Stage 1	Rise >/= 0.3 mg/dl within 48 h or >/= 1.5–1.9 × reference range^*^	No	>0.5 ml/kg/h and < /= 1 ml/kg/h
Stage 2	Rise >/= 2–2.9 × reference range^*^	No	>0.3 ml/kg/h and < /= 0.5 ml/kg/h
Stage 3	Rise >/= 2.5 mg/dl	Dialysis	< /= 0.3 ml/kg/h

*The modified neonatal criteria include only changes in serum creatinine and other accepted criteria shown above*.

*AKI, acute kidney injury*.

* *Reference is lowest previous value excluding day 0 and day 1 of life*.

The AWAKEN (Assessment of worldwide acute kidney injury epidemiology in neonates) study retrospectively assessed a multinational, multicentre cohort of infants in 24 neonatal intensive care units (NICU) and aimed to understand the epidemiology of AKI in neonates with the new unified definition (1). In this study, there was an overall incidence of 29.9% of AKI. In the preterm group, ELBW infants had a higher incidence of AKI (48%) compared to neonates born at 29–36 weeks gestation (28%). Additionally, any episode of AKI in neonates increased the risk for mortality by three-fold compared to age-matched controls without an AKI.

Askenazi et al. prospectively observed 923 infants born <28 weeks gestation from birth until hospital discharge as part of the Preterm Erythropoietin Neuroprotection Trial (PENUT) and found that 38% had at least one episode of AKI and 18.2 % had an AKI defined as severe (16). The rates of AKI increased significantly as birth GA decreased: from 9.4% at 27–28 weeks gestation to 27.8% at 24–25 weeks gestation. Both lower birth weight and GA were independently associated with higher rates of AKI after controlling for the other variable. However, the rates of AKI for those born <24 weeks completed gestation were not quoted.

Neonatal AKI using the nKDIGO definition is additionally associated with increased morbidity, specifically longer length of stay, progressive chronic kidney disease (CKD), hypertension, poor neurocognitive outcome, and increased risk of infection and sepsis as a consequence of impaired innate immunity (17–24). Despite acceptance, many limitations to this definition remain and are even more challenging in the ELBW population. Specifically, the persistence of maternal creatinine during the first 3 days of life, reduced GFR, and creatinine absorption at the level of the proximal tubules make it difficult to interpret sCr in this population (5, 25).

Additionally, using the nKDIGO sCr definintion to define AKI requires a baseline sCr for comparison. This remains problematic as there is no baseline steady state in neonates in the first day of life, and the sCr is expected to rise and then decrease physiologically in the postnatal period in preterm infants (15, 26). The natural history of creatinine level in neonates, especially those born extremely prematurely is variable (26). In a healthy neonate sCr at birth may reflect maternal sCr but over the following days will reflect innate renal function (15). In preterm infants sCr will increase in the first 3 days before decreasing (26–28). The peak will be highest in ELBW infants and occurs later with a slower descent than in their term counterparts (26–28).

The nKDIGO definition also assumes that there are a series of sCr levels available for comparison, and that these levels presumed to be the “baseline” have not been affected by early pathological processes e.g., early onset sepsis and hypotension. However, iatrogenic anemia in ELBW infants from repeated serum leads to careful consideration of risk and benefit with each needed blood draw, often limiting the number of sCr levels available for comparison (29). AWAKEN, and others, have shown that incidence of AKI in these populations directly correlates with the number of sCr checked (1, 30).

The diagnosis of AKI by urine output (UOP) measurement is especially challenging in the NICU. Use of a bladder catheter in premature infants is anatomically difficult secondary to small size and increases the risk of structural damage and susceptibility to infection. Accurate weight of nappies to estimate UOP is limited by evaporation in the humidified incubator and the risk of mixture with stool (5). Defining abnormal UOP in this population has also proven challenging. The often quoted <0.5 ml/kg/h r over an 8-h period is extrapolated from the pediatric population and has never been validated in neonates (31, 32). Additionally, due to the lower urinary concentration ability in neonates and variability across GAs, it has been suggested that the threshold of concern for low UOP in neonates should be higher than that of older children (33).

The limitations to available scoring systems as discussed above, has led to suggestions that z scores or centile charts of renal function based on gestational age norms, rather than fixed values would be more appropriate to measure kidney function of preterm infants in the first weeks of life (34).

## Etiology

The vast majority of AKI in the ELBW population is thought to be multifactorial and can be pre-renal, intrinsic (renal), and obstructive (post-renal) failure. Extremely preterm infants may be more vulnerable to AKI secondary to factors related to prematurity including maternal medications, difficulty maintaining fluid homeostasis and changes in haemodynamic states, hypoalbuminemia, and the use of unavoidable nephrotoxic medications.

### Factors Related to Prematurity

Factors unique to the preterm neonate in the prenatal and perinatal period include maternal medications and conditions during pregnancy and the impact of prematurity on renal function. Maternal indomethacin is additionally used antenatally as a form of tocolysis in threatened preterm labor and is associated with neonatal renal insufficiency. Antenatal steroids, such as betamethasone, used for the maturation of the respiratory system, have been shown in retrospective studies to decrease post-natal AKI and lower sCr levels in preterm infants (35). The signifigant effects that antenatal exposure to both of these medications has on the GFR of the neonate was shown by Van Den Anker et al. (36) in a study of 147 preterm infants between 23 and 37 weeks gestational age (mean 30.2 weeks), comparing GFR of infants who were exposed to one, both or neither of these medications antenatally. Infants exposed to indomethacin only had a significantly lower GFR than those exposed to neither, while no difference was seen between the group receiving both medications and those receiving neither.

Post-natal contributors to AKI that are unique to the preterm infant include increased susceptibility to infection, haemodynamically significant patent ductus arteriosus (PDA), and the need for long-term central vascular access. The contribution of hemodyamic instability and medications are addressed separately.

Systemic to pulmonary shunting created by a haemodynamically significant PDA can cause a “ductal steal” with resultant pulmonary overload and worsening respiratory status and compromise perfusion to other organs including the kidneys (37, 38). The use of neonatologist performed point of care ultrasound, including doppler flow in the abdominal aorta can aid in diagnosis of decreased flow in the renal vessels which may cause later kidney injury. Near-infrared spectroscopy (NIRS) is a similar tool and a non-invasive method of measuring renal tissue oxygenation continuously (39). However, to date no standardized guideline for their use exists and neither ultrasound parameters or NIRS monitoring have not been shown to predict development of AKI (40).

Many preterm neonates require central access through the umbilical vessels for the provision of medications and nutrition, measurement of blood pressure and minimization of phlebotomy. Despite the use of heparinised infusates to decrease risk of thrombosis, and close attention to positioning of umbilical lines, migration of umbilical catheters can lead to renal artery and vein thrombosis ultimately contributing to AKI in premature infants (41, 42). Careful positioning of umbilical venous (UVC) and arterial catheters (UAC) by the clinician is necessary to decrease the risk of thrombosis. An UAC should be placed in a high position (tip above the diaphragm, T6–T9) as lower positioning has been linked to an increased risk of thrombosis (43). UVC tip placement should be at the junction of inferior vena cava and the right atrium and should be at T9–T10. X-ray is generally used to confirm adequate position. Serial ultrasound has also been used to ensure adequate position (44). Renal vein thrombosis is rare and presents as frank haematuria, flank mass, hypertension and thrombocytopenia and can lead to AKI. Often this is a clinical diagnosis. The addition of renal artery doppler improves the sensitivity of ultrasound in detecting thrombosis, but it remains technically difficult, and the only findings are enlarged echogenic kidneys. Management of thrombosis through anti-coagulation may be required acutely to mitigate the long-term consequences, in the case of more severe or propagating thrombosis. Hypertension secondary to thrombosis in the renal vessels may necessitate treatment with anti-hypertensive medications. Severe thrombosis can potentially need dialysis in the short and longer term. Assessment of hypertension in the infant who has had umbilical lines, with or without a preceding AKI should include ultrasound with doppler for this reason.

### Volume Depletion, Hemodynamics, and Ischaemic Injury

Hypotension is common in ELBW infants with almost 25% having at least one episode of documented hypotension during their hospitalization, with older reports suggesting, an even higher incidence (45, 46). Hypotension can occur for various reasons in the neonatal population-hypovolaemia, distributive shock in sepsis or necrotising enterocolitis and cardiac failure being potential culprits. Hypotension can lead to renal hypoperfusion and ultimately contribute to the development of AKI.

Intravascular volume depletion caused by hypovolaemia, sepsis with capillary leak or decreased oncotic pressure secondary to hypoalbuminemia is a common contributing factor of neonatal AKI (2, 33). Hypovolaemia can have numerous causes but those specific to the preterm neonate include increased insensible losses from fragile skin and the need for humidified incubation for temperature control. A secondary effect of excess fluid loss and volume depletion in the ELBW infant is hypernatremia, which itself can be associated with AKI. The etiology of hypernatremia in preterm infants in the first week is more often dehydration rather than excess sodium administration (47). Dehydration can occur in the context of high insensible water losses, high urine output and/or negative fluid balances. Extremely premature infants have a higher total body water content, allowing increased vulnerability to dehydration secondary to high insensible losses.

In the preterm neonate multiple factors both pre and postnatally can predispose to sepsis including premature prolonged rupture of membranes, chorioamnionitis (the most common cause of spontaneous preterm birth), the necessity of indwelling intravenous catheters for the delivery of medication and nutrition and the fragility of skin predisposing an infant to nosocomial infection. Ischaemic injury to the kidney from low cardiac output states may also lead to renal injury.

Polyuria in the ELBW infant can contribute to hypovolemia and subsequently AKI. It is typically defined as urine output >4 ml/kg/h, but can be as high as 6 ml/kg/h in ELBW neonates (48). Immature nephrons have a poor capability to concentrate urine leading to free water clearance and the development of polyuria. An osmotic diuresis secondary to hyperglycamia in the sick neonate, with free water being lost in the urine along with sugar, is a potential cause of polyuria. Excipients used as part of intravenous medications such as mannitol use in paracetamol preparations can also contribute. Therefore, careful attention to maintain adequate hydration while avoiding pathologic fluid accumulation is vital.

There are multiple rare causes of polyuria in the neonate which the provider should consider when determining etiology. Neonatal Bartter syndrome is a rare renal tubular disorder of that manifests as severe polyuria associated with antenatal polyhydramnios and often preterm delivery (49). This will often have associated other metabolic derangements. Central diabetes insipidus can also cause polyuria (50). Other rarer conditions with polyuria and potential for AKI include pseudohypoaldosteronism, cystinosis, and congenital adrenal hyperplasia Hyperoxaluria is another cause of neonatal AKI, it is important to measure an oxalate level if no other explanation is noted for renal failure.

### Medication(s)

Nephrotoxic medications are a common cause of AKI in the NICU (33). These often-unavoidable medications include antibiotics, antivirals, and antifungals for empiric management of infection risk after delivery and the treatment of both early and late onset sepsis. Aminoglycosides e.g., gentamicin and glycopeptides e.g., vancomycin used for the treatment of bacterial sepsis, liposomal amphotericin for fungal infection, acyclovir in viral infections are the most commonly seen. It can be difficult to reliably distinguish the effect of the underlying sepsis and secondary AKI, from the nephrotoxic effects of the treating medications in these cases, however vancomycin and gentamicin levels monitoring in the context of AKI is vital. When vancomycin or gentamicin levels are elevated a review of dosing is required including reducing dosing frequency/prolonging interval and increase testing of trough levels. If GFR is particularly low the dose may need to be adjusted. Electronic medical record flags in context of prescribing nephrotoxins, a rising sCr or AKI can be useful. Education and engagement of all members of staff to the importance of therapeutic drug level monitoring plays a vital role in prevention of AKI.

The use of ibuprofen and indomethacin for the closure of a PDA can also cause kidney injury. Indomethacin, a prostaglandin synthase inhibitor, used in this population both for PDA closure and the prophylaxis of intraventricular hemorrhage, has been seen to cause renal injury in 40% of those treated, although this is usually rapidly reversible with no long-term consequence (33, 51). This includes a 56% reduction in urinary flow rate, a 27% reduction in GFR, and a 66% reduction in free water clearance (33, 52). Ibuprofen, a non-steroidal anti-inflammatory, causes renal vasoconstriction via blockage of cyclooxygenases and prostaglandin synthase. It has been shown in both randomized trials and meta-analyses to have less of an effect on renal function than indomethacin (53). More recently paracetamol has been used in the treatment of PDA. Concerns have been raised regarding a link between paracetamol and autism/neurodevelopmental delay in cohort studies of prenatal exposure and liver injury (54). However, this is not seen in post-natal cohort studies of paracetamol use (55–58). Multiple studies comparing PDA treatment are ongoing with results awaited to ensure the safety and efficacy of paracetamol as a treatment.

Intravenous immunoglobulin (IVIG), used in the treatment of hyperbilirubinaemia can cause an osmotic insult caused by the high sucrose contents of most forms of IVIG, although very rarely used as a treatment strategy in ELBW infants, and far more commonly employed in term and near-term infants (2, 59, 60). In contrast, the use of caffeine citrate, a methylxanthine, used in the treatment of apnoea of prematurity, has been shown in retrospective review of the AWAKEN cohort to reduce incidence and frequency of AKI in ELGAN, a 3-fold reduction in the incidence of AKI and an 8-fold reduction in stage 3 AKI in the first week after birth (61). Methylxanthines are adenosine antagonists that act via A1 and A2 receptors including receptors present in the kidney. Other methylxanthines including theophylline and aminophylline, used in other neonatal pathologies such as neonatal encephalopathy have been shown to prevent AKI and improve renal function but are not used in the ELBW infant (62).

The Baby NINJA (Nephrotoxic Injury Negated by Just-in-Time Action) quality improvement (QI) initiative attempted to decrease neonatal AKI caused by nephrogenic medications (63). Carried out over 24 months (including an 18-month sustainability period) in a level IV (non-delivery NICU), the initiative comprised of an automated screening report of patients with high nephrotoxic medication exposure (defined as ≥3 nephrotoxic medication within 24 h or ≥4 calendar days of an intravenous (IV) aminoglycoside). In infants with high nephrotic exposure meeting study criteria, pharmacists recommended daily SCr during the exposure period and for 2 days post exposure/episode of AKI. The team then discussed alternative medication and management strategies to decrease AKI risk. No specific recommendations on medication use were mandated by the protocol. This QI initiative showed a reduction in high nephrotoxic medication exposures from 16.4 to 9.6 per 1,000 patient-days (*P* = 0.03), reduction in percentage of nephrotoxic medication-AKI from 30.9 to 11.0% (*P* < 0.001), preventing 100 AKI episodes during the 18-month sustainability era. The Baby NINJA project shows that closer monitoring of SCr to diagnose AKI and drawing attention to the side effects of commonly used NICU medications, large improvements can be made and sustained over time.

## Assessment and Management

The assessment and management of neonatal and ELBW AKI is likely to differ across institutions because of a lack of published guidelines and paucity of research in this area. Until recently AKI was thought to be a reversible problem associated with acute illness. With newer evidence of the short- and longer-term consequences of AKI, the importance of accurate diagnosis and management of AKI in the NICU is becoming increasingly recognized.

Infants at risk of AKI should be closely monitored—with clinical assessment, sCr levels and monitoring of UOP and fluid balance. These include, but are not limited to, infants who become acutely unwell including with sepsis and septic shock, are undergoing surgery, have known haemodynamically significant PDA's, with polyuria or oliguria, infants being treated with known nephrotoxic medications as detailed above or requiring inotropic support. Frequent assessment of renal function is necessary to diagnose and manage AKI appropriately and mitigate the long-term effects, as seen in the data from the AWAKEN study (1).

### Assessment of AKI—Current and Future

Current definitions of neonatal AKI require the assessment of sCr (with changes from baseline) and assessment of UOP. However, sCr rise is only notable with significant renal dysfunction. Therefore, newer more sensitive renal biomarkers have been validated in children and adults. More recently several biomarkers have been shown to be useful in preterm neonates measured either in serum or urine and may have a role in predicting longer-term renal outcome and renal disease stratification (64).

Neutrophil gelatinase-associated lipocalin (NGAL) belongs to the lipocalin superfamily and has been found in activated neutrophils and other cells including renal tubular cells. NGAL is an early predictor of AKI and is released from the distal tubule (65). The proximal tubule releases urinary kidney injury molecule-1 (KIM-1), liver-type fatty acid binding protein (L-FABP), insulin-like growth factor-binding protein-7 (IGFBP-7), and tissue inhibitor of metalloprotease-2 (TIMP-2) while the loop of Henle secretes uromodulin (UMOD) (66). Projects to develop a kidney tissue atlas such as the Kidney Precision Medicine Project, will allow the use of biomarkers to assess specific areas of renal dysfunction and guide therapy for AKI. NGAL has excellent prediction of AKI on day 1 of life with an area under the curve (AUC) of 0.91 for stage I and 0.92 for Stage II/III (67). Urine epidermal growth factor (EGF) had a similar predictive profile for AKI stage I with AUC 0.97 and Stage II/III 0.86 (67).

Urinary biomarkers show variation with GA in stable healthy preterm infants. For example, EGF and UMOD increased with advancing GA reaching levels of term infants by 3 months (68). Huynh et al. described the median, 95th and 99th percentiles, and range of pooled UNGAL values were 5, 50, 120, and 2–150 ng/mL, respectively. Higher levels with greater variability in uNGAL were found in female vs. male infants (69). Askenazi also found significant variation of renal biomarkers with GA in preterm infants and noted that NGAL, osteopontin (OPN) and beta-2-microglobulin (B2mG) when corrected for sCr levels were independently associated with GA (70). Lavery et al. also found uNGAL was easily obtained in premature infants and correlated significantly with both birth weight and GA (71). Despite these findings, as these biomarkers are compared against sCr, it is unclear if this inverse relationship is reflective of “normative” ranges or a higher incidence of subclinical injury.

Urinary annexin A5, uNGAL and protein S100-P levels were also promising biomarkers for early, accurate prediction of AKI in preterm infants as assessed on proteomics (72). Using metabolomics uNGAL and KIM-1 correlated with the severity of kidney injury in preterm infants without significant morbidities or sepsis (73). Calbindin, Collagen IV, Fatty Acid Binding Protein 1, Alpha Glutathione S-Transferase, Serum IFN-γ-induced protein 10, KIM-1, Osteoactivin, Renin, trefoil factor 3, tissue inhibitor of metalloprotease-2, α-1-Microglobulin, Albumin, Clusterin, Cystatin C, EGF, Lipocalin-2/NGAL, and OPN were measured at 3 days and 3 weeks after birth (74). Markers of glomerular and tubular function increased with GFR over the first 3 weeks of life with no changes in cystatin C (74).

In preterm infants' sepsis and the presence of a PDA have also been associated with changes in renal biomarkers. uNGAL may be impacted by sepsis as it correlated with C reactive protein (CRP) and clinical severity but also correlated well with AKI (75). In septic neonates both serum and urinary NGAL rose significantly in septic infants as well as those with AKI (76). Urinary N-terminal pro B-type natriuretic peptide (NT-proBNP), NGAL, and Heart-type fatty acid binding protein (H-FABP) may predict hemodynamic relevance of PDA in very low birth weight infants (77). In asphyxiated preterm infants and NGAL is predictive of renal injury (78). Higher NGAL levels (≥82 ng/mL) at birth but not neutrophil count have been association with the development of bronchopulmonary dysplasia [gestational-age adjusted odds ratio (OR) = 37.45 (3.08–455.49), *p* < 0.01] (79).

Serum cystatin C has been evaluated as a more accurate and useful biomarker of acute kidney injury in the ELBW infant. Studies in the premature (80) and ELBW (81) population have attempted to establish normative reference ranges for cystatin C. The study by Armagil et al. (80), assessing 108 infants with a mean gestation of 32.5 ± 2.6 weeks, mean weight 1,830 ± 590 g found a serum cystatin C range of 1.25–2.84 mg/L on day 1 of life, with a signifigant decrease by day 3, likely due to maturation of innate renal function. Creatinine was found to correlate negatively with gestational age. Cystatin c was independent of gestational age as well as gender, birth weight and hydration status. Demirel et al. (81) assessed cystatin C in 113 ELBW infants, finding a mean of 1.77 ± 0.38 mg/L on day 1, falling to 1.61 ± 0.37 ml/L on day 3. There was significant correlation seen between maternal and infant creatinine, but not maternal creatinine and cystatin C, suggesting that cystatin C may help overcome the issues of maternal creatinine on neonatal creatinine in the diagnosis of early AKI.

Therefore, the use of renal biomarkers could improve the early diagnosis of AKI in preterm infants and guide response to therapy and potential impact of nephrotoxic medications.

### Evaluation of Etiology

#### Renal USS in AKI

Renal ultrasound should be performed in all neonates with AKI. This may be technically challenging in ELBW infants, and limited published data exist on sonographic assessment of renal size in this population (82). Ultrasound can identify congenital anomalies of the kidney and urinary tract, which may include bladder outlet obstruction amenable to urological intervention, as well as other intrinsic renal abnormalities. Echogenicity is a non-specific finding, often observed in the context of AKI, although can be difficult to interpret in very small or preterm infants, as what constitutes a normal degree of echogenicity in this group, is not well-defined (83, 84). Doppler flow should be evaluated to ensure patency of the renal vasculature, particularly in children who have had umbilical vessel catheterisation.

#### Medication Review

The assessment of etiology of AKI in ELBW infants should include a thorough evaluation of medications likely to be causing or contributing to kidney injury. This review should include an evaluation of each medication and whether a change to medication type/dosage/frequency could decrease the impact on renal function without compromising the treatment of the underlying condition. As shown by the Baby NINJA initiative, drawing attention to the nephrotoxic effects of commonly used NICU medications, can drastically decrease rates of AKI in a sustainable fashion (63).

### Treatment Strategies and Management of Short-Term Consequences

#### Fluids and Fluid Balance

The primary aim of fluid management in neonates with AKI is the maintenance of euvolemia (17). The key to achieving this is careful and frequent assessment of the volume status of the infant. Clinical assessment, including vital signs, change in weight, serum electrolytes, fluid balance, particularly accurate recording of UOP are important.

When examining the fluid balance, the contribution of medications, “flushes” and feeds to total intake needs to be recognized. It is also important to consider insensible losses which can be substantial in extremely preterm and low birth weight infants. Insensible losses increase with decreasing GA and birthweight and are most significant in the early postnatal days. The contribution of evaporative water losses through immature skin is a significant factor which reduces as the infant matures and the skin epithelializes. At 24–25 weeks GA the trans-epidermal water losses have been estimated to be as high as 140 ml/kg/day in a 1,000 g infant at 50% humidity; this can be reduced significantly by increasing the humidity of the environment (85, 86). Although a general estimate of insensible losses can be made based on the body surface area using the calculation 300–400 ml/m^2^/day in older children, unfortunately it is not possible to be as prescriptive in small and preterm infants given the multiple contributing factors at play and thus careful monitoring of fluid balance, as described above, is essential (87).

Fluid resuscitation may be necessary in the clinically hypovolaemic infant to restore circulating volume and optimize renal perfusion. The response to fluid challenge (10–20 ml/kg over 1–2 h) should be monitored closely and care to avoid precipitating fluid overload is crucial as it has associations with poorer outcomes including increased mortality and intraventricular hemorrhage, especially in the first days of life (88, 89).

The management of fluid overload in the context of an oliguric AKI in ELBW infants can be challenging. Restriction of total fluid intake in an infant with high glucose requirements poses a particular challenge. Matsushita FY et al. reviewed ELBW infants (*n* = 219) and <900 g in a retrospective cohort study and found that severely fluid overloaded in the first 3 days in ICU, had higher mortality and required longer periods of ventilation. There was similar levels of confounding variables including antenatal steroid exposure between groups (90). Similar reports from neonatal intensive care, involving all gestations with a small subgroup of infants under 37 weeks, use of continuous renal replacement therapy (CRRT) indicated that those with fluid overload at the start of CRRT had worse outcomes (91). Fluid overload of >15–20% in neonates is associated with poor outcome defined as need for dialysis (91).

#### Diuretics

If the restriction of fluid is insufficient to manage fluid overload, addition of diuretics may be beneficial. Re-establishing UOP in response to diuretic therapy simplifies fluid management and may circumvent the need for renal replacement therapy. However, improving urine output with diuretic therapy does not equate to an improvement in renal function or GFR, as the mechanism of diuretics result in electrolyte and free water clearance and do not act directly on renal function (92). Frusemide is a frequently used loop diuretic in the NICU. Bolus dosing is often initially used intravenously or orally. Initial IV dosing for under 31 weeks is 0.5–1 mg/kg/dose. Oral dosing under 31 weeks tends to be 0.5–2 mg/kg/dose. Infusions of frusemide can be considered at the following dosing 0.05–0.4 mg/kg/h with limited data in ELBW infants but in renal failure higher doses are often required to stimulate a diuresis.

Bumetanide is a more powerful loop diuretic with limited literature available on appropriate dosing to be used in ELBW infants. The dose can be given intravenously or orally with an available dosing range of 0.1 mg/kg/dose on a frequency of up to 4 times per day. Best responses have been demonstrated at 0.035–0.04 mg/kg/dose (93, 94). Bumetanide can also be given as an IV infusion with a dose range of 1–10 mcg/kg/h (95). Hearing loss associated with bumetanide, noted during the NEMO (NEonatal Seizure Using Medication Off-patent) trial of bumetanide for seizure treatment in term infants with Neonatal Encephalopathy limits its utility in this group (96). Metolazone is a powerful thiazide diuretic for severe oedema and is used orally typically 0.2–0.4 mg/kg/day in divided doses with limited studies in ELBW infants (95).

#### Electrolyte Imbalances

Electrolyte derangement is common in AKI including hyperkalaemia, hyponatremia, hyperphosphatemia and hypocalcaemia. Average sodium needs for preterm infants are higher than term infant, on day 1–2 they can require 3 mmol/kg/day, on day 3 between 6 and 12 mmol/kg/day and day 7 between 4 and 8 mmol/kg/day (97). When hypernatremia (defined as serum sodium >150 umol/l) occurs, the causes is often inadequate free water administration, and may be associated with clinical evidence of volume depletion. Optimizing the fluid intake and free water will be the first step in management (98–101). When hypernatremia occurs without volume depletion in AKI, minimizing additional delivered sodium from red blood cell transfusion, albumin infusions, parenteral nutrition, carrier solutions for other medications, is especially important in first few days of life as increased IVH are seen in the context of early hypernatremia (102).

Hyponatremia in the context of AKI is not uncommon and should be managed based on the etiology of the hyponatremia—ultimately whether the issue has arisen from too much water or too little salt. A state of water excess, as can be seen with increased anti-diuretic hormone secretion (either inappropriately or appropriately) will manifest clinically as hypervolemia, management in this context will thus focus on fluid restriction. Hyponatremia in the context of a euvolemic or hypovolaemic infant should prompt a review of the sodium intake, and consideration given to potential avenues for excess sodium loss. Infants have immature renal tubules with limited capacity for sodium and water reabsorption, thus are vulnerable to high urinary sodium losses (103). Replacement, in general, cautiously aims to increase serum sodium no more than 8–10 mmol/L in 24 h. More aggressive correction with hypertonic saline solutions, may be required in the context of acute symptomatic hyponatremia; presenting with seizures, for example, targeting an initial prompt rise in serum sodium. Studies in older children support aiming for an initial rise of 5 mmol/L over the first few hours is safe and effective (104–106).

The ability to excrete potassium is reduced in infants, particularly preterm infants. Reduced expression and function of sodium-potassium-ATPase pumps and responsiveness to aldosterone lead to higher serum potassium levels in this age group. Although preterm infants can tolerate relatively high levels of serum potassium compared to older children there is still a risk of toxicity and as such hyperkalaemia should be managed promptly and appropriately (107). Management of hyperkalaemia may include the following: use of insulin/glucose infusion to stimulated cellular uptake of potassium; use of sodium bicarbonate to correct acidosis and encourage intracellular shift of potassium; or use of frusemide to increases urinary excretion of potassium in infants with urine output (104). Potassium-containing intravenous fluids should be avoided in infants with AKI. A study of 40 preterm infants (<36 weeks) suggested the use of nebulised salbutamol may be as effective as insulin/dextrose in infants with non-oliguric hyperkalaemia with less impact on blood glucose (108).

#### Albumin

Administration of human albumin solution for the purposes of volume expansion or treatment of hypo-albuminemia has a reasonable theoretical basis, with the suggestion that improving plasma oncotic pressure could limit capillary leak and third spacing of fluid and improve intravascular volume status (109–114). Preparations are available in a range of concentrations from 5 to 20%. A meta-analysis of 90 cohort studies identified a low serum albumin as a predictor of poor outcome with some studies finding that maintaining serum albumin above 30 g/dL with albumin infusion may be of benefit. While another study found albumin administration increased urinary output particularly with the use of diuretics in the oedematous neonate (115). A Cochrane review was unable to determine whether routine use of albumin infusion in preterm neonates with low serum albumin reduces mortality or morbidity, however, only two studies were eligible for inclusion (116). Albumin administration is associated with a risk of precipitating volume overload and therefore is usually given with frusemide (117). In infants with AKI the risk of shifting fluid intra-vascularly that then cannot be excreted due to poor renal function is of particular concern and could potentially be catastrophic (118). Infants with oliguria or anuria who are being treated with albumin infusion should be discussed with nephrologists in a tertiary.

#### Nutrition and Protein Intake

Adequate nutrition for ELBW infants is one of the major determinants of long-term outcome (119). Managed volumes of allowed fluid and high volume of intra-venous medications can leave little room to provide adequate nutrition in this population, either by total parenteral nutrition or enterally with maternal or expressed breast milk. This issue is amplified in an unwell infant with an AKI who may require fluid restriction for management of the AKI as well as increased medications for management of the underlying cause of the AKI e.g., antibiotics, inotropes, sedation. One of the major challenges of managing AKI in this population is ensuring adequate nutrition is maintained while avoiding fluid overload, electrolyte imbalances and hyperglycaemia.

Parenteral glucose and lipid provision are the main source of calories for the preterm infant. In the absence of adequate calories from this source's protein sources will be utilized for energy which can in turn increase the blood urea nitrogen (BUN). Therefore, adequate nutrition is important not only for growth and development of the infant but also in the management of the AKI (120).

Protein intake is generally limited in patients with AKI and increasing blood urea nitrogen as restriction of protein in adult patients showed better outcomes (120). Protein requirement in this population to avoid growth failure and consequent poorer neurodevelopmental outcome impairment is 3–4.5 g/kg/day (121). Protein intake must at least meet the infant's basal growth requirement of 1–2 g/kg/day while striving to maintain as close to 4.5 g/kg/day without increasing BUN and therefore serum osmolarity (25, 122).

#### Dialysis

Data is scarce on the utilization of dialysis for ELBW infants with AKI but is associated with a high risk of mortality. Additionally, neonates with a higher percentage of fluid overload and higher levels sCr at RRT initiation show poorer outcomes. Information on dialysis in this group is often case report based and is not attempted in all centers.

Peritoneal Dialysis (PD) is the only option in many units and may not be possible in this size category and is limited by skin integrity. The indication for use is often refractory hyperkalaemia. PD is considered the preferred modality for neonates with AKI. PD is not feasible in patients with major abdominal surgery, severe hyperammonaemia, necrotizing enterocolitis and abdominal wall defects. Risks of leak are higher and thereafter infection when PD started for AKI soon after catheter placement. PD in ELBW infants requires the use of manual sets, as the automated machines are unable to handle the low volumes required.

It is important to differentiate neonates who need acute dialysis with likely recovery from the chronic group whom are unlikely to stop PD. In a review of the available literature, one such report from a unit with acute PD, had data available on 16 very low birth weight infants (24–30 weeks; 630–1,430 g) using the 14-gauge Arrow vascular catheter™ who had reversible challenges such as PDA, sepsis, NEC. Acute PD was successful in 12 infants for management of hyperkalaemia, fluid overload and metabolic acidosis with typical complications associated with dialysis of leak, peritonitis and hernia and 6 babies died (123). A study of 45 neonates who received dialysis for hyperammonaemia used PD (23) and continuous or intermittent dialysis (14 continuous veno-venous haemodialysis—CVVHD, 5 continuous arterio-venous haemodialysis—CAVHD, 3 haemodialysis—HD). All modalities performed similarly with no association between outcome and dialysis modality (124).

Both vascular access and available technologies for CVVHD modalities can be very challenging in this group. Continuous renal replacement therapy (CRRT) is challenging in critically ill neonates, from the perspective of vascular access to allow high blood flow rates. One of the main challenges with using CRRT circuits in smaller infants is the extra cellular volume of the circuit, and filters with bradykinin release side effects on haemodynamic stability. Most CRRT equipment available is not designed for ELBW infants but for older children and adults. Achieving accurate ultrafiltration can be difficult on CRRT in neonates. CRRT practices can be modified to fit the needs of infants and neonates, work is ongoing on development of better technology for this population. CRRT machines available for use in most intensive care units are currently approved for children >25 kg. Neonates therefore often need large prime volumes and blood primes to ensure stability.

Lee et al. studied CRRT in 34 neonates with AKI using the neonatal RIFLE (nRIFLE) criteria to describe the severity of AKI. Forty four % were between 25 and 36 weeks, and 60% of these infants were extremely or very low birth weight and mortality was 50% (91). CVVHD was used with Prisma or Prismaflex (Gambro/Baxter Int., Lund, Sweden) dialysis machines and M10 (50 ml) or HF20 (55 ml) filters and central access using 6.5-French (Fr) hemodialysis catheters. Oliguria was identified as a poor prognostic factor and was associated with mortality. CRRT was run initially without anticoagulation and heparin was started only if the initial CRRT filter was lasting under 12 hours.

New technologies are in development including the miniaturized Cardio-Renal Pediatric Dialysis Emergency Machine (CARPEDIEM) developed by Ronco et al. (125). The benefits include a reduced Extracorporeal Volume (27 ml). The CARPEDIEM also has sensitive scales for infusion and effluent bags to improve the accuracy of ultrafiltration. Another system is the Newcastle infant dialysis and ultrafiltration system (NIDUS) which has been used in infants as low weight as 800 g. Coulthard et al. described 9 infants (lowest weight 1.8 kg) using single lumen vascular access without the need for blood priming and the extracellular volume was <10 ml (126).

Askenazi et al. worked on an adaptation of The Aquadex FlexFlow system (Gambro) with ECV of 33 ml but issues arose with hypothermia, ultrafiltration accuracy and anticoagulation with heparin and 6 of 12 infants survived (127). However, their weights were higher in this group- median of 3.4 kg, and minimum noted to be 2.7 kg. Focusing on the ELBW group, there is very rare experience with acute dialysis regularly and development of reliable safe tools for CRRT will need to be observed in development.

When deciding on offering renal replacement therapy in this group, open discussion with the MDT and the family needs to include risks benefit balance regarding the chance of recovery from AKI, reasons why recovery may not occur and other risk factors such as prognosis of the underlying condition, neurological risks, risks associated with multiorgan dysfunction, risks of dialysis and anticoagulation and long-term prognosis (128). There is a background chance of the AKI evolving into a chronic or end stage renal disease (128, 129). Some families may prefer not to attempt dialysis and center-specific counseling and outcomes will be relevant.

### Management of Long-Term Consequences

As with many areas of neonatology, as the survival of the smallest infants increases, the longer term effects of their prematurity and treatment in NICU becomes more relevant. There is little work assessing the long-term health effects of the ELBW population as they grow into adulthood, especially related to kidney injury in the neonatal period. International collaboration incorporating the long-term effects of prematurity are required to further evaluate this and improve both early and late management. Better understanding of the functional maturation of GFR (130) of the preterm infant as they grow will also aid management.

#### Blood Pressure

Infants with AKI are almost twice as likely to develop hypertension (131). Hypertension in the context of AKI is generally a product of volume overload and/or activation of the renin-angiotensin-aldosterone system, leading to systemic vasoconstriction. Hypertension is often under-recognized in preterm and low birth weight infants. One study identified that hypertension was diagnosed in 1.8% of infants admitted to NICUs, but a further 3.7% were retrospectively classified as having undiagnosed hypertension (131). The major contributors to under-recognition of hypertension in this population include difficulties obtaining reliable and accurate measurement of blood pressure in small infants and uncertainty around the definition of hypertension in this population.

Normative data is challenging to compile, as several factors contribute to blood pressure (BP) in small and preterm infants (132–134). Dionne et al. have published BP percentiles based on post-menstrual age (135). Accurate measurement of blood pressure is crucial. Intra-arterial measurement via an umbilical or peripheral catheter and an appropriately positioned transducer (at the level of the heart), is the gold standard (136). The accuracy of non-invasive BP measurement can be improved using an appropriately sized cuff (internal bladder at least 80% of the circumference of the arm) and ensuring that the infant is restful and not stimulated–moving, crying, or feeding (137–139).

Recommendations on the use of both intravenous and oral antihypertensive agents in neonates are available, however given the complexity of the area, and limited evidence for use of agents in preterm infants specifically, clinical renal expertise is valuable (137, 140). Intravenous infusions are required for acute severe hypertension and care must be taken to avoid rapid fluctuation in blood pressure (141). Issues with absorbtion in the premature gut may also lead to a need for intravenous rather than oral agents. Angiotensin converting enzyme inhibitors should be avoided in AKI and are also not recommended infants <44 weeks post menstrual age due to potential risk of precipitous and profound drop in blood pressure, hyperkalaemia, or AKI (137, 142, 143). In the context of significant or difficult to manage hypertension consideration should always be given to a renovascular lesion, and prompt radiological investigation arranged.

#### Chronic Kidney Disease

Prematurity at birth is a risk factor for the development of early chronic renal disease. Urinary NGAL levels were higher in ex- ELBW in adulthood subjects compared to controls. In addition, hematic asymmetric dimethylarginine (ADMA) concentrations is associated with adverse cardiovascular events and cardiac death in this population and early chronic kidney disease may contribute to atherosclerosis and increase the risk of future adverse cardiovascular events (144). AKI in any child infers a subsequent risk of long-term renal sequelae (145). As such infants with an episode of AKI in the early neonatal period should be followed up with annual surveillance for the development of hypertension or proteinuria.

## Conclusions

AKI is relatively common in ELBW infants and is associated with significant long-term consequences and morbidity in adulthood. Early recognition, monitoring and follow up is required and careful management regarding potentially nephrotoxic medication and fluid balance.

## Author Contributions

AB, CC, MS, and EM contributed original work to the original draft of the article. EM and CS were reviewed and edited the original draft and prepared a final draft with AB. All authors critically revised the manuscript, agree to be fully accountable for ensuring the integrity and accuracy of the work, and read and approved the final manuscript.

## Conflict of Interest

The authors declare that the research was conducted in the absence of any commercial or financial relationships that could be construed as a potential conflict of interest.

## Publisher's Note

All claims expressed in this article are solely those of the authors and do not necessarily represent those of their affiliated organizations, or those of the publisher, the editors and the reviewers. Any product that may be evaluated in this article, or claim that may be made by its manufacturer, is not guaranteed or endorsed by the publisher.
